# Exudates of *Picea abies*, *Pinus nigra*, and *Larix decidua*: Chromatographic Comparison and Pro-Migratory Effects on Keratinocytes In Vitro

**DOI:** 10.3390/plants11050599

**Published:** 2022-02-23

**Authors:** Thomas Goels, Elisabeth Eichenauer, Ammar Tahir, Paul Prochaska, Franziska Hoeller, Elke H. Heiß, Sabine Glasl

**Affiliations:** Division of Pharmacognosy, Department of Pharmaceutical Sciences, University of Vienna, Althanstraße 14, 1090 Vienna, Austria; thomas.goels@univie.ac.at (T.G.); elisabeth.eichenauer@univie.ac.at (E.E.); ammar.tahir@univie.ac.at (A.T.); a1009982@unet.univie.ac.at (P.P.); franziska.hoeller@univie.ac.at (F.H.); elke.heiss@univie.ac.at (E.H.H.)

**Keywords:** Pinaceae exudates, *Picea abies* balm, *Picea abies* resin, *Pinus nigra* resin, *Larix decidua* balm, fingerprint comparison, in vitro re-epithelialization

## Abstract

Balms and resins of *Picea abies*, *Larix decidua*, and *Pinus nigra* are traditionally used to treat wounds. Three chromatographic techniques differing in separation capacity and technical demands were employed to distinguish among these plant exudates. A TLC method was established for fingerprint comparison, providing a quick overview of a large number of samples at low cost. HPLC-DAD (RP18) and UHPSFC-DAD (Torus 2-Picolylamin), hyphenated to ESI-MS, represented orthogonal chromatographic systems with high separation performance. The developed methods allow for the separation and detection of major and minor constituents belonging to different compound classes (phenyl carboxylic acids, lignans, diterpene resin acids). The qualitative compositions of the diterpene resin acids, the main compounds in the exudates, were comparable in all three genera. Differences were detected in the distribution of hydroxylated diterpene resin acids, pinoresinol, and hydroxycinnamic acids. The three tested chromatographic systems with varying demands on lab equipment offer appropriate tools for the quality assessment of *Picea abies*, *Larix decidua*, and *Pinus nigra*. The extracts were furthermore tested at three different concentrations (10 µg/mL, 3 µg/mL, and 1 µg/mL) for boosted re-epithelialization, a crucial step in the wound-healing process, in an in vitro HaCaT keratinocyte-based scratch assay. Lysophosphatidic acid (LPA, 10 µM) and extracts of several medicinal plants well known for their wound-healing properties (birch, marigold, St. John’s wort, manuka honey) were used as positive controls. *Picea abies* and *Pinus nigra* showed concentration dependency; significant activity was measured for *Larix decidua* at 3 µg/mL.

## 1. Introduction

**Figure 1:**
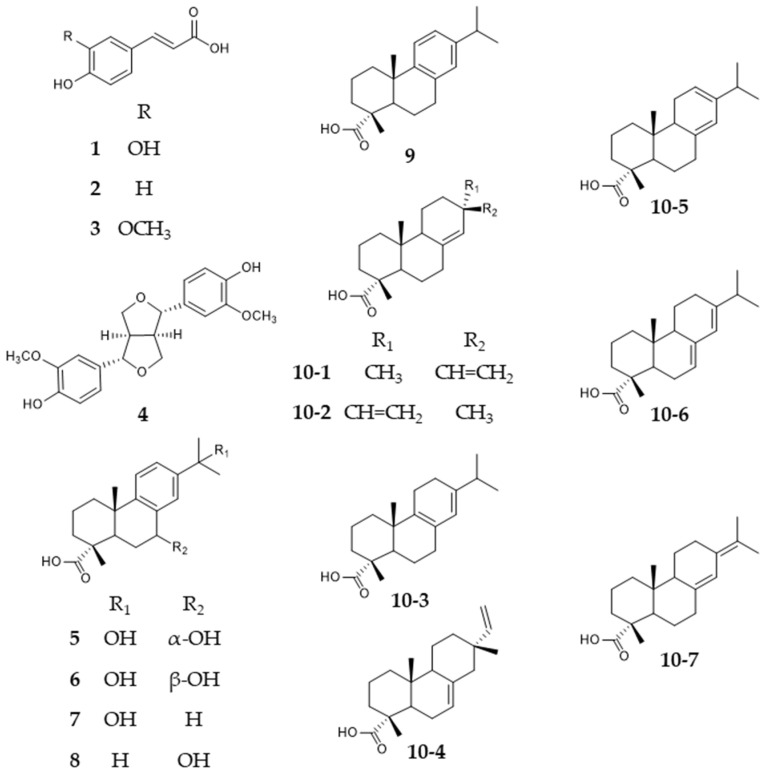
Hydroxycinnamic acids (**1**–**3**), lignans (**4**), and diterpene resin acids (**5**–**8**, **9** (300 Da), **10-1**–**10-7** (302 Da)) contained in Norway spruce balm. **1**—caffeic acid, **2**—p-coumaric acid, **3**—ferulic acid, **4**—pinoresinol, **5**—7α,15-dihydroxydehydroabietic acid, **6**—7β,15-dihydroxydehydroabietic acid, **7**—15-hydroxydehydroabietic acid, **8**—7-hydroxydehydroabietic acid, **9**—dehydroabietic acid, **10-1**—pimaric acid, **10-2**—sandaracopimaric acid, **10-3**—palustric acid, **10-4**—isopimaric acid, **10-5**—levopimaric acid, **10-6**—abietic acid, **10-7**—neoabietic acid.

Skin diseases are globally increasing [1]. The Global Burden of Disease study, based on health data from over 195 countries, reported skin and subcutaneous diseases to have grown from 493 million patients in 2005 to 605 million in 2015 [2]. Impaired wound healing occurs as co-morbidity of obesity, diabetes, autoimmune disease, bedsore, etc., burdens health care systems worldwide with tremendous costs [3,4,5,6]. There is a critical need for advanced wound management and additional efficient and broadly accessible therapies. One viable approach would be to make use of traditional knowledge about “wound healing” and provide scientific evidence for the benefits of traditional remedies [7,8]. In this line, the excretion products of different members of the Pinaceae family have been used for centuries in traditional European medicine to treat wounds. This study focused on *Picea abies*, *Pinus nigra*, and *Larix decidua*, as they play an important role in Austrian folk medicine [9,10,11] with their balms (kneadable, sticky mixtures of resins and essential oil [12]) and brittle resins (which do not contain essential oil [12]) processed to lipophilic ointments for acute, chronic, and infected wounds. Scientific research and literature comparing both the wound-healing properties and the phytochemical composition of the exudates of *Picea abies*, *Pinus nigra*, and *Larix decidua* are relatively scarce. Whereas there are studies about Norway spruce balm [13,14,15], data on European larch and black pine are still missing. Norway spruce balm consists of hydroxycinnamic acids such as ferulic acid, lignans such as pinoresinol, and hydroxylated and non-hydroxylated diterpene resin acids as main compounds [13] (see Figure 1). A monograph of this balm was included in the Austrian pharmacopoeia in 2016, followed by a monograph for a lard-based salve in 2019. The monograph envisions a simple TLC check for Norway spruce balm, but lacks sufficient analytical parameters for quality control. The first aim of this paper was to establish chromatographic methods at three different sophistication levels to facilitate analysis in simply-equipped as well as high-tech laboratories. An improved TLC method was developed including R_f_-values and assignment of prominent bands for correct identification. HPLC-DAD and UHPSFC-DAD-MS methods enabled the separation of highly similar structures such as diterpene resin acids with only minor structural differences (Figure 1). Secondly, based on these techniques, the resins of *Picea abies* and *Pinus nigra* and the balms of *Picea abies* and *Larix decidua* were compared. Thirdly, referring to the potential wound-healing claim, the balms/resins were tested in an HaCaT keratinocyte based in vitro assay for enhanced re-epithelization. Lysophosphatidic acid (LPA, 10 µM) and extracts of several medicinal plants well known for their wound-healing properties (birch [16,17], marigold [18,19,20], St. John´s wort [21,22], manuka honey [23,24]) served as references in these experiments.

## 2. Results

### 2.1. Chromatographic Comparison of Pinaceae Exudates

As already mentioned, the exudates of Pinaceae trees are complex mixtures of different substance classes. The first aim was to establish a simple and cheap TLC method, as well as high performance techniques achieving the separation of the structurally highly-related diterpene resin acids. The latter involves resin acids of the abietic- and pimaric-types sharing the same molecular weight of 302 Da and differ only in the position of one double bond and a side chain (compounds **10-1**–**10-7**, Figure 1). Hydroxylated resin acids (**5**–**8**) and dehydroabietic acid (**9**) are easier to separate due to their greater difference in structure and molecular weight [13]. The obtained fingerprints should facilitate comparison and differentiation of the exudates on a qualitative level.

#### 2.1.1. Thin-Layer Chromatography (TLC)

**Figure 2:**
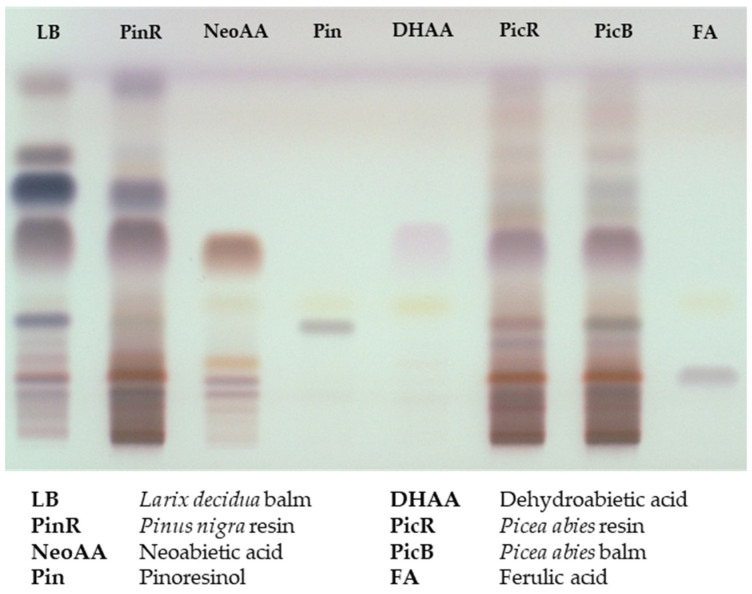
TLC comparison of Pinaceae exudates and reference substances after derivatization with anisaldehyde/sulphuric acid in daylight (stationary phase: silica 60 F_254_; mobile phase: chloroform-methanol-trifluoroacetic acid (97 + 3 + 0.1)).

TLC is a planar chromatography technique and provides a time- and cost-effective phytochemical fingerprint of multiple samples at the same time [25]. It should allow the comparison of the qualitative composition of the three exudates. We analyzed the extracts together with selected representatives of different substance classes present in resins and balms of Pinaceae as references i.e., ferulic acid (**3**, hydroxycinnamic acid), pinoresinol (**4**, lignan), dehydroabietic acid (**9**), and neoabietic acid (**10-7**, diterpene resin acids). Figure 2 shows the resulting TLC plate after derivatization with anisaldehyde/sulphuric acid solution.

The balm and resin of *Picea abies* revealed comparable fingerprints on the TLC plate; the resin of *Pinus nigra* showed a prominent additional band at R_f_ 0.7. The most significant differences at R_f_ 0.7–0.8 and R_f_ 0.2–0.3 appeared in the balm of *Larix decidua*. No separation was achieved for the resin acids. Neoabietic acid **10-7** and dehydroabietic acid **9**, shown as representative standards in Figure 2, and all other resin acids (**10-1**–**10-6**, data not shown) appeared as one single large spot at R_f_ 0.5.

#### 2.1.2. High-Performance Liquid Chromatography-Diode Array Detection/Mass Spectrometry (HPLC-DAD/MS)

**Figure 3:**
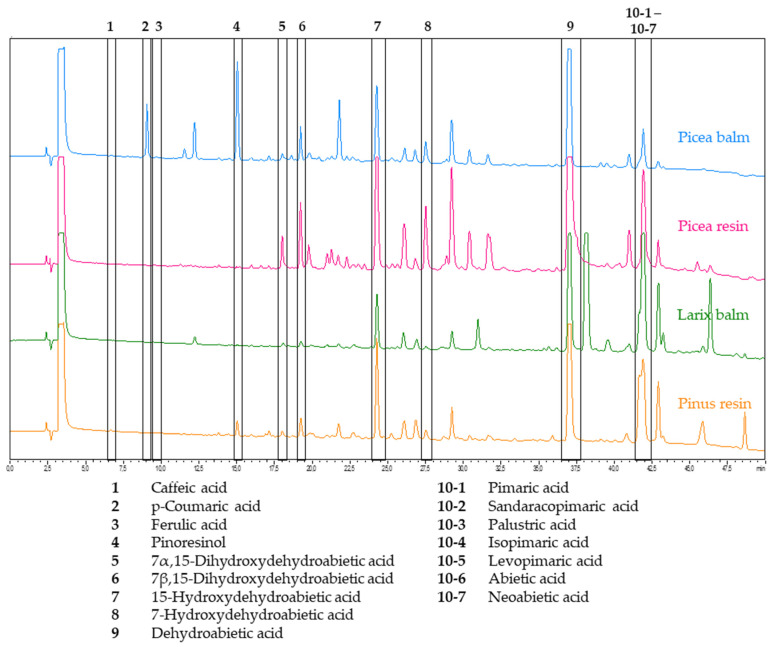
HPLC-DAD chromatograms of each Pinaceae exudate at 190 nm (stationary phase: RP18e; mobile phase: water + 0.1% formic acid, acetonitrile + 0.1% formic acid); the peaks were assigned by co-chromatography with the pure reference substances.

Subsequent to planar normal phase TLC, a high-performance liquid chromatography (HPLC) method with a reversed phase RP18 column was developed. By means of co-chromatography with pure reference substances, it was possible to assign the most prominent peaks. In Figure 3, the HPLC-DAD chromatogram (detection at 190 nm) of each exudate is depicted, including the annotations of the used reference substances. Rough semi-quantitative estimations were deduced from the obtained peak sizes.

The hydroxycinnamic acids (**1**–**3**) caffeic acid, p-coumaric acid, and ferulic acid were minor constituents of the analyzed samples: only p-coumaric acid was detected and it appeared solely in the balm of *Picea abies*. The lignan pinoresinol (**4**) was present only in Norway spruce balm and, to a considerably lesser extent, in black pine resin. The four hydroxylated resin acids (**5**–**8**), 7α,15-dihydroxydehydroabietic acid, 7β,15-dihydroxydehydroabietic acid, 15-hydroxydehydroabietic acid, and 7-hydroxydehydroabietic acid, were detected in all exudate samples. The peak of any of the four hydroxylated resin acids was lowest in the balm of *Larix decidua*. Dehydroabietic acid (**9**) was present in all samples, and eluted approximately five minutes before all other diterpene resin acids with a molecular weight of 302 Da. The other acids (**10-1**–**10-7**) eluted together as one peak. Even the use of different gradients of the mobile phase could not achieve separation of the resin acids with a molecular weight of 302 Da by HPLC-DAD with RP18 as a stationary phase.

Making use of an alternative UHPLC instrumentation, hyphenated to ESI-TOF-MS, using a Kinetex RP18 column with 2.6 µm particle size and mixtures of acetonitrile and methanol as organic components of the mobile phase confirmed the HPLC-DAD results. Unknown compounds were characterized by *m*/*z* values and sum formulas (see Table S1). Experiments were conducted to differentiate the overlapping resin acids **10-1**–**10-7**: Different MS parameters were adjusted to potentially provoke differences in the fragmentation patterns, but none of the attempts led to any successful annotation of compounds **10-1**–**10-7**.

#### 2.1.3. Ultra-High-Performance Supercritical Fluid Chromatography-Mass Spectrometry (UHPSFC-MS)

Supercritical CO_2_ used as a mobile phase provides a highly lipophilic solvent and is widely used in the analysis of nonpolar plant constituents. By adding organic modifiers, the dissolving capacity and the elution of more polar substances can be adjusted, which makes ultra-high-performance supercritical fluid chromatography (UHPSFC) a potent technique in the analysis of plant extracts containing compounds with a wide range of polarities [26].

**Figure 4:**
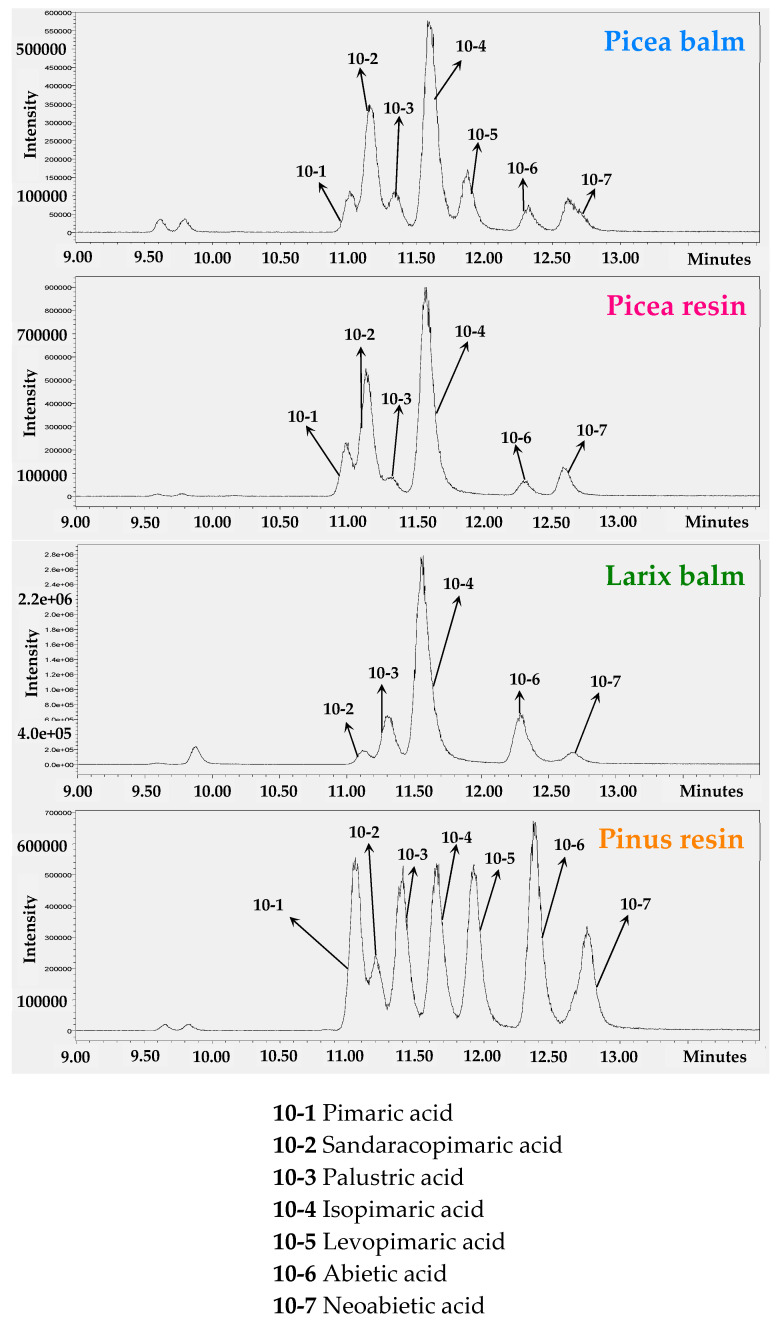
Ion chromatograms at 301 Da in negative mode of the Pinaceae exudates gained by UHPSFC-MS (stationary phase: Torus 2-Picolylamin; mobile phase: CO_2_, ethanol); the peaks were assigned by co-chromatography with the pure reference substances.

Using a Torus 2-Picolylamin separation column and ethanol as modifier, it was possible to separate the diterpene resin acids with a molecular weight of 302 Da. The mass spectra acquired in the negative mode were used and the ion chromatograms at 301 Da were extracted. Figure 4 shows the respective ion chromatograms at 301 Da of each exudate.

The four analyzed samples differed in the relative content of the various diterpene resin acids. Only the balm of *Picea abies* and the resin of *Pinus nigra* contained all of the seven resin acids, although in different quantities. Levopimaric acid (**10-5**) was not detected in Norway spruce resin and larch balm. Furthermore, pimaric acid (**10-1**) has not been detected in larch balm.

### 2.2. Adaption of the Bio-Assay

In the next step, the plant exudates were tested in vitro for enhanced re-epithelialization of a gap in a monolayer of human adult low-calcium high-temperature (HaCaT) keratinocytes, as published previously [13,27]. Positive controls included lysophosphatidic acid and extracts of birch, marigold, St. John´s wort, and manuka honey—all well known for their wound-healing properties. The handling of the assay demanded adaptions regarding the solubility of the extracts and the creation of the cell-free gap, in particular.

#### 2.2.1. Lipophilic Extracts in Aqueous Cell Systems

The application of cell-based assays in aqueous medium as in vitro models for skin biology often causes solubility to be a bottleneck for tests of lipophilic extracts or compounds, and requires for compromises in the experimental setup. In order to maximize the solubility of the nonpolar conifer extracts, the solubilizers Tween 80 (0.05%–5%), isopropyl myristate (3 and 5%), Poloxamer 188 (0.01–10%), and soy lecithin (1–10%) were tested. They were dissolved in water at the given concentration ranges. The stock solutions of the extracts were prepared in DMSO (100 mg/mL) and diluted with the respective solubilizer-containing solutions. However, the substances were either unable to solubilize the nonpolar extracts (isopropyl myristate, Poloxamer, soy lecithin) or appeared to be toxic for the cells at the tested concentrations. Tween 80 caused cell lysis after 24 h of exposure. Consequently, the use of solubilizers was avoided to rule out any impact on the cells other than from the extracts. All extracts were dissolved in DMSO (stock solutions) and diluted with medium, with the exception of Manuka honey, which was directly dissolved in medium. Thus, the test concentrations needed to be selected so that they did not yield any obvious precipitates or lipophilic aggregates when added to the culture medium (1–10 µg/mL).

#### 2.2.2. Generation of Cell Gap

The establishment of uniform cell gaps emerged as a challenge during the first experiments. The scratch assays followed the protocol published by Liang et al. [27], and scratches were initially introduced using a 200 µL pipette tip, which is frequently described in literature [28,29]. This procedure requires extreme caution, as one wants to disrupt only the cell layer by evenly sized scratches, but not to damage the coating of the cell culture vial (which would hinder the attached cells from migrating or proliferating). Various alternatives to the classical “pipette tip scratch” were sought that could guarantee reproducible sizes of the cell free area and intact surface coating at the same time. Inserts in the form of silicone cell spacers from Ibidi performed well, but were for single use only. Attempts for re-use failed, since the high temperature during sterilization caused increased adhesiveness of the silicone and again, destruction of coating during removal. Finally, the use of cross-shaped commercially available plastic inserts from LUX^®^ tools was successful. They were re-usable and weighted down with small metal weights to allow formation cell-free gaps in a growing cell monolayer, with acceptable variation in size with concurrent intact coating.

### 2.3. Results of Tested Extracts in the Bio-Assay

All plant extracts exhibited a tendency to enhance gap closure in the keratinocyte monolayer compared to solvent control cells, which can most likely be attributed to increased cell proliferation or migration.

#### 2.3.1. *Larix decidua*, *Picea abies*, *Pinus nigra*

**Figure 5:**
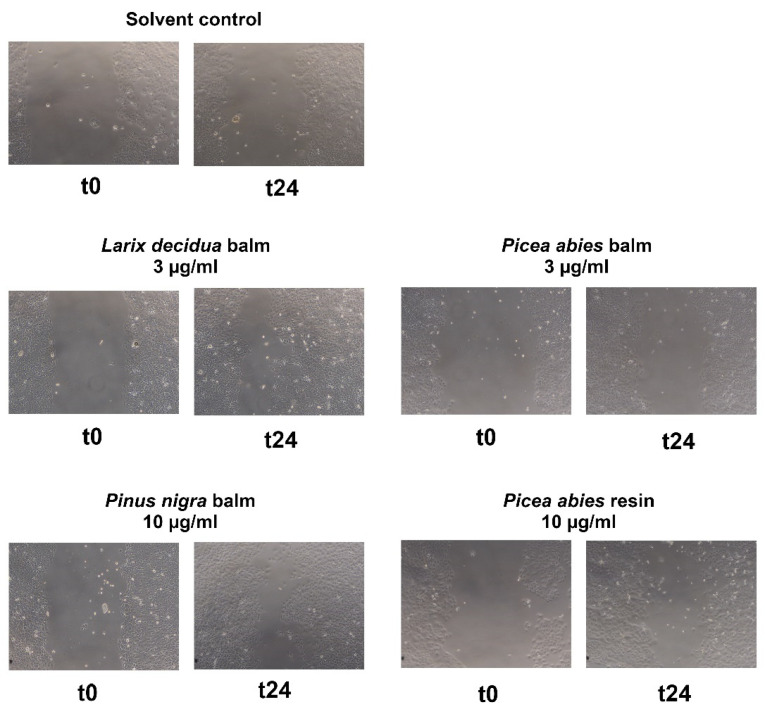
Effects of conifer resins and balms at different concentrations on scratch wound healing of HaCaT cells at timepoint t0 and after 24 h (t24).

The extracts of *Picea abies* and *Pinus nigra* elicited a moderate and concentration-dependent decrease in cell-free area, but statistical significance (see Table 1). The balm of *Larix decidua* boosted re-epithelialization at 3 µg/mL (+26.0%, statistically significant, see Figure 5) but resulted in negative values at 10 µg/ missed mL (see Table 1). This points to cytotoxic/cytostatic effects at the higher concentration, which could be confirmed in the resazurin conversion and crystal violet assay (data not shown).

#### 2.3.2. Herbal Positive Controls

**Figure 6:**
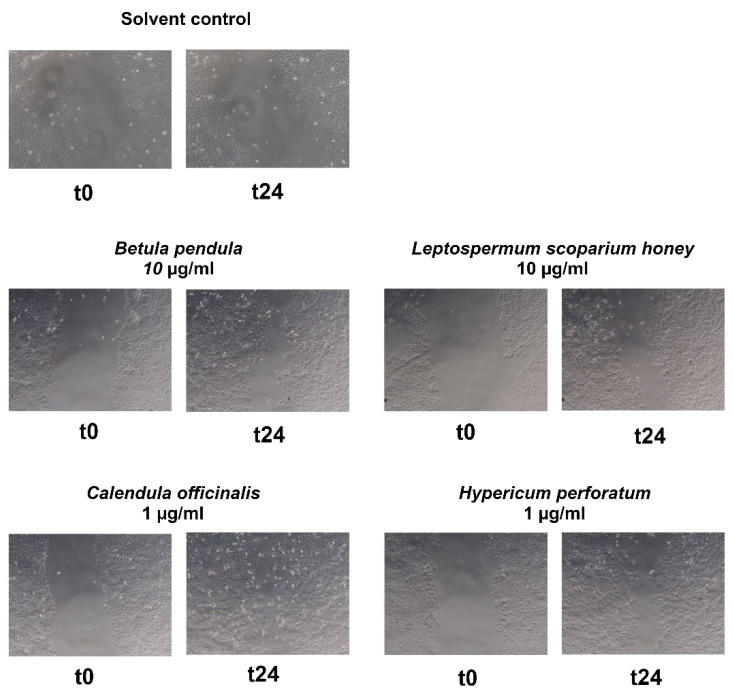
Effects of herbal positive controls at different concentrations on scratch wound healing of HaCaT cells at timepoint t0 and after 24 h (t24).

In order to better rank the activity of the tested exudates, we also ran the assay with other traditionally used wound-healing agents. *Betula pendula* (10 µg/mL: +41.6%), *Calendula officinalis* (1 µg/mL: +62.1%), and *Leptospermum scoparium* honey (Manuka honey) (10 µg/mL: +42.0%) demonstrated statistically significant differences to the vehicle control (see Figure 6). The extract of *Hypericum perforatum* showed moderate activity on keratinocytes (see Table 2). Concentration-dependency was shown for birch and Manuka honey.

The extract of St. John’s wort did not show significant activity on keratinocytes, even though this plant is known as one of the most potent medicinal plants to cure burns. It even revealed negative values at 10 µg/mL (see Table 2). This might be due to the different spectrum of constituents in the tested aqueous alcoholic extract (70%) compared to the traditionally prepared oily extract, or due to targets in the wound-healing process other than re-epithelialization. Marigold showed good activity, but exhibited an unexpected concentration-dependent decrease in activity. Assessment of HaCaT metabolic activity and cell mass after treatment with the extracts indicated an inhibitory impact of the *Calendula officinalis* tincture on cell viability (data not shown). This may provide a possible explanation for the low performance of the plant extract, especially at higher concentrations.

## 3. Discussion

The balms of *Picea abies* and *Larix decidua* and the resins of *Picea abies* and *Pinus nigra* were investigated side by side for their phytochemical composition by three different developed analytical methods and for their ability to enhance re-epithelialization in vitro. This study complements and significantly contributes to the currently existing picture in this area of research, as most studies on the phytochemical composition and wound-healing properties of conifer balms have been conducted with *Picea abies* [13,14,15]. The essential oil of cones and needles of various *Pinus* species [30,31] and extracts from the bark of *Pinus brutia* [32,33] also showed promising in vivo activity with regard to wound healing. However, the excretions of *Pinus nigra* and *Larix decidua* have been barely scientifically studied regarding their wound-healing effect. Moreover, the bioactive constituents have rarely been identified and the (relative) composition of the bark extracts remained largely unknown, which leaves a knowledge gap in research on the wound-healing properties and constituents of different *Pinus* species. In addition, GC [34,35] and HPLC [36,37] are well-established, whereas SFC protocols for these exudates have not been published so far.

The balms and resins were analyzed via three chromatographic techniques and compared semi-quantitatively regarding their composition. Detailed descriptions of patterns and differences are discussed in Section 2.1.1, Section 2.1.2 and Section 2.1.3. The value of TLC is represented by the comparison of chemical fingerprints and the throughput of a high number of samples within a short time. The TLC pattern of *Larix* clearly differed from the other exudates by three additional bands with different colors. *Pinus* and *Picea* resembled each other, but could also be distinguished by one additional prominent band occurring only in *Pinus*. The two high-performance techniques outperformed TLC regarding separation power and provided additional structural information about single compounds. The chromatograms obtained by HPLC-DAD covered various substance classes and revealed more or less minor differences between the exudates with the exception of *Larix*. The separation of the highly similar resin acids required employment of UHPSFC-MS. The relative amounts of the resin acids varied, and again showed *Larix* as easy to distinguish from *Pinus* and *Picea*. In parallel, an in vitro assay was performed to evaluate the proliferation- and migration-enhancing potential of the exudates in keratinocytes. Tested extracts showed moderate activity, and balm and resin extracts performed comparably. The only significant activity was seen with *Larix decidua* at a concentration of 3 µg/mL, which boosted gap closure by 26%, which was more potent than birch extract at the same concentration. Other conifer extracts showed partly good and concentration-dependent mean values, but also high standard deviations. The latter may still be caused by solubility issues (despite the lack of obvious precipitates). Moreover, wound healing is a complex process that involves various cell types and signaling pathways, and is usually divided in four phases. Blood clotting (Stage 1: hemostasis) occurs momentarily after an injury. Immune cells then infiltrate the wound site and cause a local inflammation (Stage 2: inflammation). In the proliferation phase (Stage 3: proliferation), keratinocytes and fibroblasts fill the wound crater with cells by migrating to the injured site and engaging in increased cell division. In this phase, new blood vessels and extracellular matrices also are formed. Maturation marks the final step of wound healing (Stage 4: remodeling), during which collagen is remodeled and scars are formed [38]. Therefore, although keratinocytes play an important role in Stages 3 and 4, and their proliferation and migration are popular readouts for assessing wound-healing properties, this assay can only capture a limited set of affected targets and may miss many others. The extracts should therefore be subjected to complementary in vitro or even in vivo models in the future.

Concerning the link between constituents and observed bioactivity, the content of hydroxylated and non-hydroxylated resin acids seems to correlate with enhanced re-epithelialization. In line with this assumption, pimaric acid (**10-1**), isopimaric acid (**10-4**), dehydroabietic acid (**9**), and combinations of isolated hydroxylated diterpene resin acids (**7** and **8**) have already displayed a significant effect in the employed bio-assay [13]. Moreover, relevant signaling pathways for wound healing are affected by abietic and dehydroabietic acid, including the TNF-α/forkhead box O1 (FOXO1), transforming growth factor-β1 (TGF-β1)/Smads, or MAPK (ERK and p38) pathways [39,40]. High concentrations of isopimaric acid may, however, counteract re-epithelialization (as seen with 10 µg/mL of the *Larix* extract or in [13]). As the extract of *Larix* was the only one to show significant bioactivity in this study, its composition might be of particular interest. As seen in Figure 4, it is characterized by a unique pattern of diterpene resin acids, in which isopimaric acid is predominant over the other acids, which are either present in minor amounts or completely missing. Moreover, in the HPLC-DAD and TLC analyses, *Larix* showed additional, unidentified peaks (e.g., peak at 38 min: *m*/*z* 312 (C_21_H_28_O_2_), and 46.0 min: *m*/*z* 388 (C_24_H_36_O_4_); see Table S1) and exclusive regions at Rf 0.2–0.3 and 0.7–0.8, respectively, that could be of importance for bioactivity. Future research must concentrate on unambiguous and detailed dissection of active principles and underlying mode of action.

## 4. Materials and Methods

### 4.1. Plant Material

The spruce balm, which has a kneadable texture and a whitish to brown color, was collected from Norway spruce (*Picea abies* (L.) H. Karst., Pinaceae, engl. Norway spruce, ger. Gewöhnlich-Fichte) in a forest near Sauerfeld, Lungau, Austria (47°07′15.0″ N, 13°52′50.0″ E). It was identified by Prof. Johannes Saukel, Division of Pharmacognosy, University of Vienna (BN. LU01). Spruce resin, which in contrast to the balm, has a typically amber color and a very sticky consistency, was obtained by removing it from spruce wood boards originating from trees grown in eastern Austria. Larch resin (*Larix decidua* Mill., Pinaceae, engl. European Larch, ger. Europa-Lärche) from Carinthia was purchased as a commercial product (BN. 124101, Schusser OG). Black pine resin (*Pinus nigra* J. F. Arnold, Pinaceae, engl. European black pine, ger. (Österreichische) Schwarzföhre) was collected in 2016 at Parapluieberg in Perchtoldsdorf, Lower Austria (48°07′02.6″ N, 16°14′00.6″ E) and identified by Prof. Johannes Saukel, Division of Pharmacognosy, University of Vienna (BN. PinNig01).

Birch bark (*Betula pendula* Roth, Betulaceae, engl. Silver Birch, ger. Hänge-Birke) was supplied by Kottas Pharma GmbH (BN. KLA30556). St. John’s wort (*Hypericum perforatum* L., Hypericaceae, engl. perforate St. John’s-wort, ger. Echt-Johanniskraut) and marigold (*Calendula officinalis* L., Asteraceae, engl. pot marigold, ger. Garten-Ringelblume) were purchased as tinctures in a local pharmacy (Tinctura Hyperici (70% ethanol) 1:5, BN. 20,171,400 FA003026, Gatt-Koller; Tinctura Calendulae (70% ethanol) 1:5, BN. 15063711, Caelo). MediHoney (BN. 1734, Dermasciences) containing pure manuka honey (honey of *Leptospermum scoparium* J. R. Forst. & G. Forst., Myrtaceae, engl. Manuka, ger. Südseemyrte) without any pharmaceutical additives was bought in a local pharmacy.

Scientific plant names were evaluated by using Medicinal Plant Names Services [41]. German names correspond to the excursion flora of Austria, Liechtenstein, and Switzerland [42], and English names were acquired from Wikispecies [43].

### 4.2. Preparation of Extracts

#### 4.2.1. *Picea abies*, *Larix decidua*, and *Pinus nigra*

As indicated in the monograph of the Austrian Pharmacopeia [44] for Norway spruce balm, the exudates of all three conifers were dissolved in acetone. Solid particles were filtered and the solvent was evaporated to give the corresponding extracts for the stock solutions in DMSO.

#### 4.2.2. *Betula pendula*

A betulin-enriched birch bark extract was prepared according to Kuznetsova et al. In brief, 10.0 g of birch bark were suspended in 300 mL ethanol 96% in a round bottom flask. Twenty percent aqueous KOH solution (14 g in 70 mL) was added and the whole solution was heated to 80 °C in a water bath for 8 h. The hot mixture was filtered and left for crystallization overnight in the fridge. 544.6 mg of betulin-enriched extract were gained [45] and used for the preparation of the stock solutions in DMSO.

#### 4.2.3. Hypericum Perforatum and Calendula Officinalis

The solvent of the respective tinctures was evaporated, and the residues were used for the preparation of stock solutions in DMSO.

#### 4.2.4. Manuka Honey

The honey of *Leptospermum scoparium* was weighed directly in an Eppendorf tube and dissolved with medium, since it was poorly soluble in DMSO. This solution was freshly prepared for every experiment.

### 4.3. Chromatographic Methods

The chemical fingerprints and the resin acid compositions of *Picea abies*, *Pinus nigra*, and *Larix decidua* were investigated via TLC, HPLC-DAD/MS, and UHPSFC-MS.

#### 4.3.1. Thin-Layer Chromatography (TLC)

For analytical TLC experiments, Silica 60 F_254_ TLC plates (average particle size 9.5 to 11.5 μm, aluminum sheets with fluorescence indicator, Merck KGaA, Darmstadt, Germany) were used. The samples of Pinaceae exudates were dissolved in acetone to a concentration of 10 mg/mL. As reference substances for the substance class of diterpene resin acids, neoabietic acid and dehydroabietic acid were used (both dissolved in acetone, neoabietic acid to a concentration of 0.5 mg/mL, dehydroabietic acid to 1 mg/mL). Pinoresinol (as representative of lignans) was dissolved in methanol to a concentration of 1 mg/mL; ferulic acid (as representative of hydroxycinnamic acids) was also dissolved in methanol to a final concentration of 2.5 mg/mL. Five microliters of each sample solution were applied by a CAMAG Automatic TLC Sampler 4 (ATS4). Development was done in the CAMAG Automatic Developing Chamber (ADC2) with chloroform-methanol-trifluoroacetic acid (97 + 3 + 0.1) as mobile phase. The CAMAG TLC Visualizer was used to interpret and photograph the developed and dried plates under white light and at wavelengths 254 nm and 366 nm. The constituents showed improved visibility after derivatization with anisaldehyde/sulphuric acid solution, which was applied with the help of the CAMAG Chromatogram Immersion Device (III) (CAMAG, Muttenz, Switzerland).

#### 4.3.2. High-Performance Liquid Chromatography-Diode Array Detection/Mass Spectrometry (HPLC-DAD/MS)

For HPLC analysis, two different instrumentations were used. For detection via diode array detector, Shimadzu instrumentation (Shimadzu Degasser DGU-20A 5, Shimadzu Auto Sampler SIL-20AC HAT, Shimadzu Communications Bus Module CBM-20A, Shimadzu Liquid Chromatograph LC-20AD, Shimadzu Column Oven CTO-20AC, Shimadzu Diode Array Detector SPD-M20A, Shimadzu Lab Solutions Software, Shimadzu, Kyoto, Japan) was employed. An RP18 column (LiCrospher 100 RP18e, 5 μm, 250 mm × 4 mm, Merck KGaA, Darmstadt, Germany) was used as stationary phase. Two solvents served as the mobile phase: double distilled water + 0.1% formic acid (solvent A) and acetonitrile + 0.1% formic acid (solvent B). Elution of the analytes was done with a gradient elution, starting with 15% solvent B rising to 95% solvent B within 45 min (rate 1.77%/min) at a flow of 1 mL/min.

For detection via mass spectrometer, an ultra-high-performance liquid chromatography instrumentation (EXIONLC AD SYSTEM (AB Sciex, Darmstadt, Germany)) was used. It was equipped with a reversed-phase RP18 column (Kinetex; 2.1 mm × 15 cm, 2.6 μm, RP18 100 Å, Phenomenex, Aschaffenburg, Germany). Mobile phase A (H_2_O/FA, 100:0.02) and mobile phase B (ACN/MeOH/FA, 80:20:0.02) were degassed prior to their usage. A 45 min binary gradient with flow rate set to 350 μL/min was applied as follows: 0−2 min, 5% mobile phase B; 2−30 min, 5−85% mobile phase B; 30–40 min, 95% mobile phase B; 40–45 min re-equilibration with 5% mobile phase B). Five microliters of each sample (1 mg/mL) were injected followed by a blank injection to ensure proper column washing and equilibration. Mass spectrometric detection was performed using the turbo ion source ESI X500 QTOF mass spectrometer (AB Sciex, Darmstadt, Germany), 500 °C heater temperature, ion source gas1 set to 30 psi, ion source gas2 set to 30 psi, curtain gas set to 45 psi. −4.5 KV/+5.0 KV spray voltages were applied to achieve negative/positive ion mode ionization. TOFMS scans were performed with an *m*/*z* range from 50 to 1500. TOF MS/MS scans of the three most abundant ions were fragmented at 35 V CES (collision energy spread), 0.1 accumulation time, and −80 V declustering potential.

#### 4.3.3. Ultra-High-Performance Supercritical Fluid Chromatography-Mass Spectrometry (UHPSFC-MS)

For the analysis of the diterpene resin acid composition, high-performance supercritical fluid chromatography coupled with mass spectrometry was utilized. An Acquity ultra-performance convergence chromatography system (UPC^2^) from Waters was used as instrumentation, consisting of a sample manager (sample temperature: 8 °C), convergence manager, binary solvent manager (flow: 1 mL/min), isocratic solvent manager (10 mM ammonium formate in MeOH-H_2_O (95 + 5); flow: 0.6 mL/min), and a column manager (column temperature: 40 °C). For detection, a Waters Acquity QDa single quadrupole mass detector (Waters Corporation, Milford, United States) was used with a mass range of 150–700 Da (capillary voltage positive 0.8 kV, negative 0.8 kV, cone voltage positive scan 15 V, negative scan 30 V). The mass spectra used for identification of the peaks were derived from the negative mode. As stationary phase, a Torus 2-Picolylamin (2-PIC) column (3.0 mm × 100.0 mm, 1.7 µm) was used. Supercritical CO_2_ and ethanol as co-solvents served as the mobile phase. A gradient system of the co-solvent starting from 0% to 3% ethanol within 8 min (hold for 2 min) and then to 5.5% within 5 min was applied. The end of the gradient was indicated by a final washing step (50% ethanol for 1 min and 0% ethanol for 1 min).

### 4.4. Bio-Assay

For the stock solutions, each extract was re-dissolved in DMSO to a concentration of 1 mg/mL. Further dilutions were prepared using DMSO except for Manuka honey, which was diluted with medium instead of DMSO. The resulting sample solutions (0.3 mg/mL and 0.1 mg/mL) were further diluted 1:100 with medium to the respective final concentrations of 10 µg/mL, 3 µg/mL, and 1 µg/mL.

HaCaT cells (CLS; Eppelheim Deutschland) were maintained in DMEM medium (high glucose, phenol-red free; Lonza, Basel, Switzerland) supplemented with 10% fetal calf serum (Gibco, Dreieich, Germany), 2 mM glutamine (Lonza), 100 U/mL benzylpenicillin (Lonza), 100 μg/mL streptomycin (Lonza) at 37 °C and 5% CO_2_ in a humidified atmosphere. DMSO (BioUltra for molecular biology) was supplied by Sigma Aldrich. LPA was purchased from Santa Cruz Biotechnology, Heidelberg, Germany.

The resazurin assay and a subsequent crystal violet assay were performed prior to the “wound healing assay” to assess metabolic activity and cell mass, respectively, which allowed deductions of cytotoxicity and tolerable concentrations of tested extracts [46,47].

**Figure 7:**
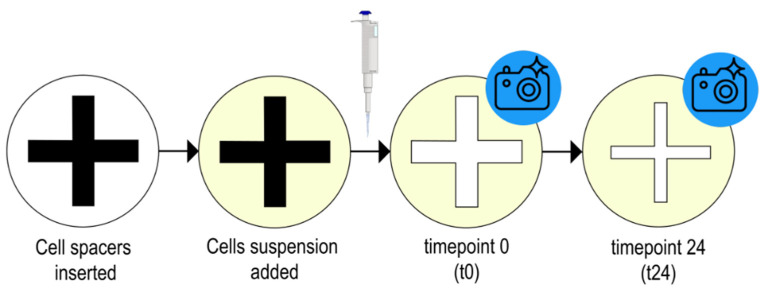
Workflow of the scratch assay on HaCaT keratinocytes.

Re-epithelialization was examined in an in vitro assay on HaCaT keratinocytes. This so-called “wound healing assay” was adapted from Liang et al. [27] (see Section 2.2.2 Induction of cell gap). In brief, cell spacers (length: 13.10 mm; width: 0.88 mm) made of plastic available in any hardware store (LUX^®^ tools, Wermelskirchen, Germany) were inserted into the wells of a 24-well plate. A suspension of HaCaT keratinocytes (1 mL of a 1.5 × 105 cells/mL solution) was pipetted into each well and the plate was incubated overnight (37 °C, 5% CO_2_). The cell spacers were removed (=t0), media was aspirated off, and debris and detached cells were removed by washing with PBS. Solutions of the plant extracts, dissolved in DMSO (BioUltra for molecular biology, Sigma Aldrich) and diluted with medium containing 0.1% fetal bovine serum to a final concentration of 1% DMSO, were applied to the wells. Figure 7 gives an overview of the workflow. As a positive control, lysophosphatidic acid (LPA, Santa Cruz Biotechnology, Heidelberg, Germany) was applied; 1% DMSO in medium containing 0.1% fetal bovine serum served as a vehicle control. Photos of the cell free gaps at the starting time (t0) and after 24 h (t24) were taken (Olympus camera PEN Lite E-PL7, Hamburg, Germany). To evaluate the cell free area of the respective gaps, Fiji, which is an image processing package containing a distribution of ImageJ combined with various Plugins [48], was utilized. For an automated analysis of each gap, the ImageJ macro “Wound Healing Tool” [49] was added to the software (variance filter radius: 24, threshold: 2–4; radius open: 4).

Two wells were tested per sample and from each well four images were taken so that, finally, a total of eight cell gaps per sample were recorded. The cell-free area at time point t24 was subtracted from the cell-free area at time point t0, which gives a measure for the obtained wound closure within 24 h. Since the camera takes pictures in a landscape format (image resolution: 4608 × 3456-pixel, aspect ratio: 1.33), the recorded four areas are inequal. For this reason, the calculations of the values resulting from the horizontal and vertical pictures were performed separately according to the formula given below: The area difference of the “vertical sample” was divided by the mean area difference of all “vertical vehicle control (VC) values”. The “horizontal values” were processed the same way. Subtraction of the value “1” sets the vehicle control to the value “0”; multiplication with “100” indicates the reduction of the cell-free area in percentage normalized to the vehicle control. The final mean value of the respective sample was calculated from these normalized values [13].

(1) $$\left(\frac{{\mathrm{Area}\;t0}_{\mathrm{sample}}{-\mathrm{area}\;t24}_{\mathrm{sample}}}{{\mathrm{area}\;t0}_{\mathrm{VC}}{-\mathrm{area}\;t24}_{\mathrm{VC}}}-1\right)\times 100$$

#### Statistics

The experiments were conducted at least in three independent biological replicates with eight technical replicates. Calculations were performed with Excel and for the statistical analysis, GraphPad Prism 6.01 (GraphPad Software, Inc., San Diego, United States) was used. Student’s *t* test was performed to compare the two groups; significance was defined as *p* < 0.05.

## 5. Conclusions

With this study, we provide three methods (TLC, HPLC-DAD/MS and UHPSFC-DAD/MS) for the differential analysis of exudates of three species from the Pinaceae family (*Larix*, *Pinus*, and *Picea*), and thus an important tool for the quality control of starting materials on the one hand, and for processed medicinal products on the other hand. A validated method for the quantitation of diterpene resin acids via UHPSFC-MS is under way.

All investigated balm and resin extracts could be shown to reproducibly enhance re-epithelialization in vitro, comparably to the reference extracts (birch, medicinal honey). The effect of the Pinaceae exudates may be in part assigned to diterpene resin acids. The role of hydroxylated resin acids (e.g., 15-hydroxydehydroabietic acid and 7-hydroxydehydroabietic acid) and yet unknown compounds of the conifer exudates, together with the extensive pharmacological characterization of active principles, are a promising starting point for future research.

## Figures and Tables

**Table 1:** Reduction of cell-free area (%) provoked by the plant extracts of *Larix decidua*, *Picea abies*, and *Pinus nigra* in the “wound healing assay” on HaCaT keratinocyte cells; given percentages are mean values (n = 3); * statistically significant (*p* < 0.05).

Plant (Extraction Solvent)	Concentration [µg/mL]	% Reduction of Cell-Free Area	±SEM	*p* Value
*Larix decidua* balm (acetone)	1	4.2	2.7	0.6472
	3	26.0 *	2.2	0.0294
	10	−4.1	14.1	0.8352
*Picea abies* balm (acetone)	1	−5.6	3.4	0.6556
	3	16.7	6.9	0.2503
	10	−2.0	6.4	0.9142
*Picea abies* resin (acetone)	1	5.9	14.2	0.7610
	3	9.6	0.9	0.5265
	10	47.6	17.2	0.0815
*Pinus nigra* resin (acetone)	1	10.9	8.7	0.5202
	3	16.2	6.7	0.332
	10	38.7	17.4	0.137

**Table 2:** Reduction of cell-free area (%) provoked by the herbal positive controls in the “wound healing assay” on HaCaT keratinocyte cells; given percentages are mean values (n = 3); * statistically significant (*p* < 0.05); ** statistically significant (*p* < 0.01).

Plant (Extraction Solvent)	Concentration [µg/mL]	% Reduction of Cell-Free Area	±SEM	*p* Value
*Betula pendula* (96% EtOH)	1	18.3	12.9	0.2757
	3	20.0	16.7	0.3037
	10	41.6 *	11.3	0.0313
*Calendula officinalis* (70% EtOH)	1	62.1 *	16.0	0.0121
	3	54.9	31.7	0.1073
	10	35.0	18.9	0.1125
*Hypericum perforatum* (70% EtOH)	1	33.1	22.9	0.2072
	3	27.3	9.5	0.1244
	10	−12.4	20.2	0.6249
Honey from *Leptospermum scoparium* (H_2_O)	1	4.9	8.9	0.7724
	3	23.1	10.1	0.2181
	10	42.0 **	12.5	0.0087

## Data Availability

All figures and tables in this manuscript are original and unpublished anywhere else.

## Supplementary Materials

The following supporting information can be downloaded at: https://www.mdpi.com/article/10.3390/plants11050599/s1, Table S1: Summary of the qualitative chromatographic comparison.
