# Supplementary material for: Exudates of Picea abies, Pinus nigra, and Larix decidua: Chromatographic Comparison and Pro-Migratory Effects on Keratinocytes In Vitro
Source: Plants (Basel). 2022 Feb 23;11(5):599. doi: 10.3390/plants11050599 (PMC8912572; doi:10.3390/plants11050599)
Supplement: Supplementary file 1 [file plants-11-00599-s001.zip › plants-1575376-supplementary.pdf]

Exudates of *Picea abies*, *Pinus nigra* and *Larix decidua*: Chromatographic comparison and pro-migratory effects on keratinocytes in vitro

Thomas Goels, Elisabeth Eichenauer, Ammar Tahir, Paul Prochaska, Franziska Hoeller, Elke H. Heiß, Sabine Glasl*

Division of Pharmacognosy, Department of Pharmaceutical Sciences, University of Vienna, Althanstraße 14, Vienna, Austria
* Correspondence: sabine.glasl@univie.ac.at; Tel.: +43 1 4277 55207

Table S1. Summary of the qualitative chromatographic comparison.

Technique	Stationary phase	Mobile phase	Detection	Results
TLC (see Figure 2)	Silica 60 F_{254} aluminium sheets (9.5-11.5 µm)	chloroform – methanol - trifluoroacetic acid (97 + 3 + 0.1)	derivatization with anisaldehyde/sulphuric acid solution	➢ Pinoresinol (**4**, R_f 0.3)<br>➢ Neoabietic acid (**10-7**), dehydroabietic acid (**9**) and other diterpene resin acids (**10-1** – **10-6**): no separation by TLC (overlap at R_f 0.5)<br>➢ Ferulic acid (**3**, R_f 0.2)<br>➢ High similarity of *Picea abies* resin and balm with slight differences in color and intensity at R_f 0.3 (band corresponds to pinoresinol **4**)<br>➢ Differentiation of *Larix decidua* by two additional prominent bands at R_f 0.7 and 0.8; less intensive bands at R_f 0.2-0.3 than in *Picea* and *Pinus*<br>➢ Differentiation of *Pinus nigra* by one additional band at R_f 0.7 and a missing band at R_f 0.3 (corresponds to pinoresinol **4**)
HPLC-DAD (see Figure 3)	LiCrospher 100 RP18e, 4x250 mm, 5 µm	acetonitrile – water (both containing 0.1% formic acid) start: 15%, gradient: 1.77%/min (0-45 min), flow: 1 mL/min	Shimadzu Diode Array Detector SPD-M20A, detection wave length: 190 nm	➢ Diterpene resin acids with m/z 302 (**10-1** – **10-7**) are not separated and elute between 41.5-42.5 min<br>➢ Dehydroabietic acid (**9**, m/z 300) is well separated from the other resin acids (R_t 37.2 min)<br>➢ Hydroxylated derivatives of dehydroabietic acid (**7**, **8**) elute more than 10 min earlier than **9** (25.2 and 27.5 min)<br>➢ Dihydroxylated derivatives of dehydroabietic acid (**5**, **6**) elute more than 18 min earlier than **9** (17.8 and 19.7 min)<br>➢ Pinoresinol (**4**) elutes at 15.0 min; detectable in the balm of *Picea* and in the resin of *Pinus* (confirmation of the results from TLC)<br>➢ Hydroxycinnamic acids (**1**-**3**) elute within the first 10 minutes but are of minor importance for the composition of the exudates; only p-coumaric acid (**2**, R_t 9.4 min) is detectable in *Picea* balm<br>➢ *Larix* balm: characterized by two additional peaks with unknown identity (38.0 and 46.0 min, m/z values see below)<br>➢ *Picea* balm and resin: similar qualitative composition; *Picea* balm: additional peaks with unknown identity at 12.5 min and 22.0 min
UHPLC-ESI-MS	Kinetex RP18 Phenomenex, 2.1×150 mm, 2.6 µm, 100 Å	acetonitrile/ methanol (80+20) – water (both containing 0.02% formic acid) start: 5% isocratic (0-2 min), gradient: 2.9%/min (2-30 min), flow: 350 µL/min	AB Sciex ESI X500 QTOF peak detection: negative mode	➢ Confirmation of the HPLC-DAD results (see Figure 3) by m/z values and sum formulas<br>➢ MS-data of unknown compounds:<br>29.5 min: m/z 318 ($C_{20}H_{30}O_3$ suggestion: hydroxylated derivative of abietic acid)<br>30.3 min: m/z 318 ($C_{20}H_{30}O_3$ suggestion: hydroxylated derivative of abietic acid)<br>31.0 min: m/z 334 ($C_{20}H_{30}O_4$ suggestion: dihydroxylated derivative of abietic acid)<br>38.0 min: m/z 312 ($C_{21}H_{28}O_2$)<br>46.0 min: m/z 388 ($C_{24}H_{36}O_4$)
UHSFC-MS (see Figure 4)	Torus 2-Picolylamin, 3.0x100.0 mm, 1.7 µm	supercritical CO_2 – ethanol ethanol gradient: 0-3% (0-8 min), hold for 2 min, 3-5.5% (10-15 min)	Waters Acquity QDa peak detection: negative mode	➢ Separation of the diterpene resin acids with m/z 302 (**10-1** – **10-7**)<br>➢ *Picea* balm and *Pinus* resin: contain all seven diterpene resin acids<br>➢ *Picea* resin: no levopimaric acid (**10-5**)<br>➢ *Larix* balm: no levopimaric acid (**10-5**) and no pimaric acid (**10-1**)
